# Aneurysmal Subarachnoid Hemorrhage and Clinical Decision-Making: A Qualitative Pilot Study Exploring Perspectives of Those Directly Affected, Their Next of Kin, and Treating Clinicians

**DOI:** 10.3390/ijerph20043187

**Published:** 2023-02-11

**Authors:** Beatrix Göcking, Nikola Biller-Andorno, Giovanna Brandi, Sophie Gloeckler, Andrea Glässel

**Affiliations:** 1 Institute of Biomedical Ethics and History of Medicine, University of Zurich, Winterthurerstrasse 30, CH-8006 Zurich, Switzerland; 2 Institute of Intensive Care Medicine, University Hospital Zurich, Rämistrasse 100, CH-8091 Zurich, Switzerland; 3 Department of Health Sciences, Institute of Public Health, Zurich University of Applied Sciences, Katharina-Sulzer-Platz 9, CH-8401 Winterthur, Switzerland

**Keywords:** aneurysmal subarachnoid hemorrhage (aSAH), brain damage, semi-structured interviews, narratives, individual experiences, thematical analysis, qualitative research, decision-making, DIPEx

## Abstract

Background: Exploring the experience and impact of aneurysmal subarachnoid hemorrhage (aSAH) from three perspectives, that of those directly affected (AFs), their next of kin (NoK), and treating clinicians, is a way to support and empower others to make informed medical decisions. Methods: In a Swiss neurosurgical intensive care unit (ICU), eleven semi-structured interviews were conducted as part of a Database of Individual Patient Experiences (DIPEx) pilot project and thematically analyzed. Interviews were held with two clinicians, five people experiencing aSAH, and four NoK 14–21 months after the bleeding event. Results: Qualitative analysis revealed five main themes from the perspective of clinicians: emergency care, diagnosis and treatment, outcomes, everyday life in the ICU, and decision-making; seven main themes were identified for AFs and NoK: the experience of the aSAH, diagnosis and treatment, outcomes, impact on loved ones, identity, faith, religion and spirituality, and decision-making. Perspectives on decision-making were compared, and, whereas clinicians tended to focus their attention on determining treatment, AFs and NoK valued participation in shared decision-making processes. Conclusions: Overall, aSAH was perceived as a life-threatening event with various challenges depending on severity. The results suggest the need for tools that aid decision-making and better prepare AFs and NoK using accessible means and at an early stage.

## 1. Introduction

Definition: Aneurysmal subarachnoid hemorrhage (aSAH) ICD-11 8B01 [1] is a serious medical event associated with significant mortality rates and high survivor morbidity [2]. Epidemiology: Those experiencing aSAH are generally between 40 and 60 years old. They are relatively young and often active with no previous significant medical history. aSAH affects about 8 people out of 100,000 each year [3], of which approximately 66% are female [4]. The World Federation of Neurosurgical Societies (WFNS) scale (grades I-V) is one of several tools for prognostication [5]. The mortality rate varies between 1% and 80%, depending on the severity of the initial bleed [5]. Treatment: Initial surgical treatment aims to secure the ruptured aneurysm by endovascular coiling or surgical clipping and to treat hydrocephalus by inserting an external ventricular drain (EVD) [6]. Complications: The risk of common complications such as early rebleeding within the first 24 h [7], vasospasm and delayed cerebral ischemia [8], which tend to occur within 3–14 days after the first bleeding event [9], and possible elevated intracranial pressure [10] all make prognostication difficult. Outcome: Despite advancements in diagnostic capability and treatment options, aSAH still has high morbidity and mortality [11]. The outcomes range from death to complete rehabilitation and recovery. The consequences of aSAH, according to the ICD catalog [12], include cognitive deficits; speech and language deficits; and motor deficits. Psychosocial changes, such as shifts in personal plans and daily life, impact the quality of life of those experiencing aSAH and their loved ones [13]. The term “next of kin” (NoK) is used in this article to denote the primary contact person(s) for healthcare personnel who are potentially involved in medical decision-making. The term “loved one”(LO) is used in a broader context, including family members and non-related persons close to the affected individual. Shared decision-making: Clinical guidelines [7] provide a framework for managing those experiencing aSAH. In the shared decision-making model [14], clinicians (CLs) and patients work together to make treatment decisions, drawing on the clinician’s knowledge of the clinically relevant risks and expected outcomes and the patient’s expression of his or her personal preferences and values. When those directly affected are unable to participate in such processes due to incapacitation, clinicians turn to the next of kin and legal documents such as advance directives to determine the declared or—if unavailable—the presumed will of the patient. Thus, treatment decision-making in the event of aSAH involves several actors. It also tends to be time-pressured since interventions are time-sensitive [15]. Due to the relative youth of those who experience aSAH and its typically sudden and unexpected onset, those directly affected tend to be unprepared and often lack written advance directives. Current studies have suggested the value of artificial intelligence to improve prognoses [16] and establish real-time decision-making support [17,18,19].

During decision-making in the acute phase of aSAH, three key features characterize decision-making: (a) Ideas about quality of life are highly individual and influenced by numerous factors, such as culture, religion, family, and personal values. Moreover, ideas about quality of life often evolve after one has survived a life-threatening event such as aSAH. (b) Those directly affected and their next of kin often only become aware of the implications of their decision after some time has passed unless they already have experience or knowledge of the disease. (c) Clinicians must decide on and enact treatments quickly in emergencies.

An overview of existing research on the perspectives of those affected and their next of kin regarding the process of decision-making and the impact of aSAH reveals that such material is scarce. The affected person’s perspective one year after aSAH was explored in research that focused on perceived and expected recovery [20]. The authors describe the event of aSAH as life-changing, affecting the patient’s self-image and, by nature, allowing for only a limited ability to predict recovery. Two reviews investigate patient-reported outcome measures in aSAH. There is a scoping review [21] that shows a qualitative research gap concerning aSAH: out of about 5000 publications, only 12 studies with patient-reported outcome measures were identified, and only 3 were based on a qualitative research design. In a systematic review of patient-reported outcome measures in aSAH [22], nine articles were identified. A qualitative follow-up study [23] explored the consequences of aSAH, such as fatigue or lessened social engagement, which are not captured by conventional scores such as the Glasgow Outcome Scale Extended (GOS-E). Following aSAH, patients often have severe cognitive and communication impairments [24,25]. The neuro-psychological consequences of aSAH are likely an important factor in why so few studies exist that consider the affected person’s perspective; those directly affected are often unable to participate in retrospective studies. Such limited available research highlights the necessity of better understanding the needs of this population.

Research on the challenges facing the next of kin [26] identified gaps in supportive services. A study of the “retrospective agreement and consent” [24] of those who have experienced neurocritical injuries shows that satisfaction depends on functional outcomes. In another paper, the authors call for the need for better “predictors for good functional outcome after neurocritical care” [27]. Scientific papers on decision-making in neurocritical care mainly [28,29] refer to those affected by ischemic stroke and emphasize the need for a stroke-specific advanced directive [28]. Qualitative research on experience in stroke refers, for example, to family adaption [30] or the therapeutic itinerary [31]. Multi-perspective qualitative studies after stroke focus on the psychological and emotional needs of those affected [32], methods for preventing chronic emotional distress [33], and needs in long-term outpatient rehabilitative care [34]. Qualitative research addressing clinicians’ perspectives and that of those affected [35] highlights opportunities to improve the timely treatment of aSAH.

The experiences of treating clinicians, the affected person, and their next of kin regarding aSAH and decision-making have not yet been compared. The outcome and evaluation of medical decision-making in neurocritical care remain under-researched, particularly regarding aSAH.

The objective of this pilot study is to explore the experience and impact of aSAH in Switzerland on the informed medical decision-making process from three perspectives: affected persons (AFs), next of kin (NoK), and treating clinicians (CLs). The specific aims are:

- To gather individual insights that might be of use to others faced with similar medical decision-making from three perspectives, AF, NoK, and CL;
- To collect and present reported individual experiences in the methodological framework of DIPEx [36], as a reliable and quality-controlled open source of individual experiences;
- To better understand the decision-making process in the event of aSAH and to explore how AFs and their NoK experience this life-changing situation and what their emerging values and priorities are;
- To put these views in the context of the experiences of clinicians.

## 2. Materials and Methods

A phenomenological approach [37] was applied for this qualitative pilot study using semi-structured interviews carried out with both clinicians working in neurosurgical intensive care units in Switzerland (nICUs) and with those experiencing aSAH and their next of kin. The different subjective experiences of disease- and health-related processes were investigated. This exploratory approach enabled the identification of a broad spectrum of experiences and outcomes, revealing relevant themes.

### 2.1. Researcher Characteristics and Reflexivity

Researcher 1 (first author) is an assistant at the Institute for Biomedical Ethics and History of Medicine (IBME) at the University of Zurich (UZH) and has a master’s degree in religious studies, focused on social sciences and gender studies. In addition, she practices as a clinical nurse in the nICU at the University Hospital Zurich (USZ) with 23 years of professional experience, specializing in the ICU. As a nurse, she has an insider perspective on this specific healthcare setting and a working relationship with the interviewed clinicians. She may have cared for those affected and may have interacted with their next of kin, but she has no access to archived patient records.

Researcher 2 (last author) is an academic associate at the IBME, is experienced in qualitative research. She has 10 years clinical working experience in inpatient and outpatient neurorehabilitation as a physiotherapist. AG holds master’s degrees in Neurorehabilitation, Public Health and Applied Ethics and a PhD in Rehabilitation Sciences. She is part of the Swiss DIPEx coordinator team.

Researcher 3 (second author) is the national representative of DIPEx Switzerland and a bioethicist with 20+ years of professional experience based on a medical background.

All three of these researchers are female, white, German-speaking, and experienced in qualitative research methods.

Researcher 4 (third author) is the deputy head of the USZ nICU and a senior physician. She revised the project description and supported the research design. She is female, white, and Italian- and German-speaking and has experience in clinical assessments.

Researcher 5 (fourth author) is a postdoctoral researcher at the IBME whose research focuses on autonomy in the face of serious illness and injury in contemporary settings. She also has clinical perspective as a practicing psychiatric nurse practitioner. She is female, white, and English-speaking.

### 2.2. DIPEx Research Methodology

This pilot study is embedded into the framework of DIPEx. DIPEx is an international collaboration of researchers, clinicians, and those affected that aims to gather and make broadly accessible individuals’ experiences related to health, care, and illness, allowing for comparisons both nationally and internationally [38,39]. The Health Experience Research Group (HERG) at the Nuffield Department of Primary Care, University of Oxford, developed the methodological basis [40]. Currently, 14 countries participate in the international DIPEx network with national platforms [38]. The results of this pilot study will be uploaded to the Swiss DIPEx website [41] as planned in 2023.

### 2.3. Collaboration, Recruitment, and Inclusion Criteria

**Figure 1:**
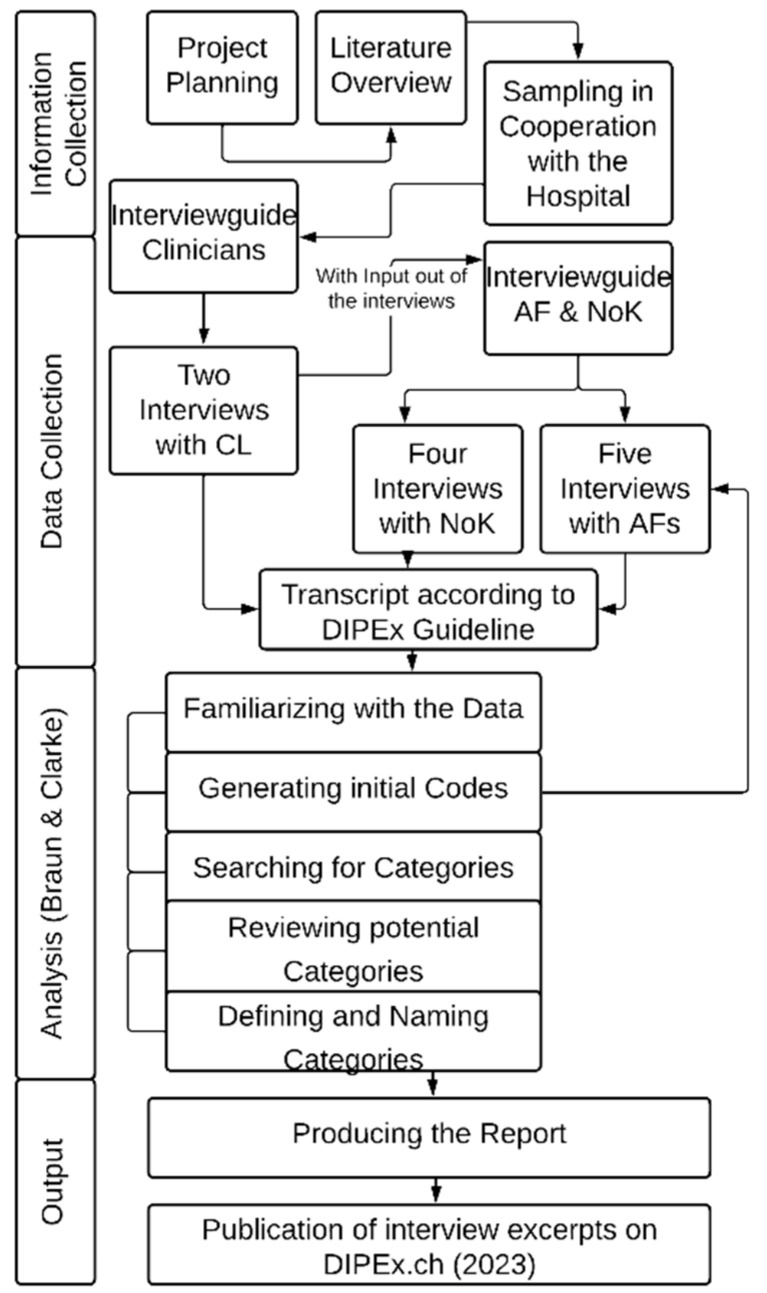
Flowchart of course of the pilot study of aSAH in Switzerland, methodical procedure.

The project was designed in collaboration with the nICU of the University Hospital Zurich (see Figure 1). Based on the DIPEx Handbook [42], the authors developed two interview guides: (a) one for clinicians and (b) one for those directly affected and their next of kin (see Table 1). The qualitative semi-structured interviews were conducted from three perspectives: (a) treating nICU clinicians, (b) those directly affected by aSAH, and (c) the next of kin. Clinicians were asked to reflect more generally on their experiences with such affected people. Interviews with those directly affected and their next of kin took place 14–21 months after the initial aSAH event.

Participants were recruited through the nICU. Two experienced nICU clinicians agreed to participate. The interviews were conducted in March 2021. Author 1 received a phone list of 28 patients with aSAH hospitalized between January and April 2020.

### 2.4. Data Collection

All interviews were conducted in German and transcribed verbatim based on established guidelines from the Swiss DIPEx project (see Figure 1). Before beginning the official study, interview instruments for both (a) clinicians and (b) those directly affected and their next of kin were tested. The instrument for version “a” was tested with an nICU nurse. The instrument for version “b” was tested with a person from the interviewer’s social circle who had undergone heart surgery. The characteristics of the participants are described in Table 2.

### 2.5. Data Analysis

All transcripts were analyzed using the software MAXQDA 2020. Thematic analysis [43,44] was employed to capture the subjective understanding of the lived experience of aSAH (see Figure 1). Due to the broad sampling (see Table 2), the wide range of outcomes, the varying age groups, and the different types of relationships, different themes, and challenges were revealed. The following example illustrates the analysis procedure in which a next of kin describes the state of the affected person:

“*So she [the AF] always came home [after work] and had to lie down immediately and was totally exhausted and couldn’t work like she did before.*”

(NoK09, 46)

This sentence was identified as relating to the themes of “fatigue”, “impaired concentration”, and “impaired thinking ability”. The category of “cognitive deficits” was identified, which these codes fit into. Ultimately, this category was placed in a broader category of “outcomes” (see Table 3).

### 2.6. Measures to Ensure Quality

First, the instruments for the interviews were carefully developed and vetted. The interview instruments were reviewed with the supervisor and with the interdisciplinary peer mentoring group “QualiZüri”, which supports qualitative research. The instruments were then cross-checked with other and established DIPEx interview guides and adjusted for consistency. A pilot study to test the instruments was then conducted with two clinicians, an affected person, and that person’s next of kin.

At six stages of the project, interdisciplinary discussions were held with advisors at the hospital and at the IBME, while informal consultations were carried out with the DIPEx team. The analysis of the results was reviewed and refined in two research workshops for peer feedback. In addition, a research diary, unpublished interim results, and memos accompanied the research process. First insights into specific cases of our data were discussed in a workshop with experts at the Excellence of Patient Care Symposium [45] and presented as a poster at the Arbeitstagung NeuroIntensivMedizin (ANIM) 2023 [46]. Interview excerpts were presented and discussed in the form of an interim results report at internal ICU training sessions.

USZ’s psychological services were involved in the project and gave advice on possible support services in case of escalation.

## 3. Results

The results are divided into three sections. The first section focuses on interviews with clinicians. The second section focuses on interviews with those directly affected and their next of kin. The third section provides a comparison of the results from section one and section two.

### 3.1. Overview of Main and Subcategories Presented from Three Perspectives

The coding of the interviews produced categories and subcategories capturing participants’ perspectives; these categories are presented in Table 3. The left side of the table presents the views of (a) the clinicians, and the right side of the table presents the perspectives of (b) those affected and (c) their next of kin.

### 3.2. First Section—Main Categories from Clinicians’ Perspectives Working with Affected Persons after aSAH

The interviews with clinicians aimed to gain insight into their daily responsibilities, emergency care experiences, views on the decision-making process, and roles in interdisciplinary teams. Five main categories emerged (see Table 3) from the material based on clinicians’ perspectives and will be explained here and illustrated with verbatim quotes from the interviewees.

#### 3.2.1. Emergency Care

The first period of illness refers to the time between the initiation of emergency care following the onset of initial symptoms and the transition to acute inpatient monitoring. The initial care goals of clinicians in the nICU were described as stabilizing, monitoring, and informing the affected person and his or her next of kin. Clinicians discussed how, as a result of aSAH, several early complications can occur, prolonging hospitalization and worsening prognosis, and emphasized that in order to achieve the best possible outcome, complications should be detected early:

“*With these patients, you simply have to recognize the signs in time beforeanything has happened and prevent it from happening*”.

(CL01, 32)

Clinicians noted that the prognosis depends not only on the degree of the initial bleeding but also on following complications, age, and comorbidities. According to the interviewees, experienced clinicians often cannot give a reliable prognosis in the acute phase, especially since computer tomography (CT) images are not always clear:

“*[…] because in the early stage [of aSAH], you can’t distinguish ischemia from edema on a CT scan*”.

(CL02, 51)

#### 3.2.2. Diagnosis and Treatment

Clinicians stated that diagnosing aSAH requires CT scanning. According to symptoms and progression, further diagnostic tests might be needed, including blood test analysis, electrocardiography, transcranial doppler (TCD), magnetic resonance imaging, and invasive multimodal neuromonitoring. Clinicians explained that treatment options depend on the location and size of the aneurysm and on the experience and availability of neurosurgeons and neuroradiologists. They reported two main treatment options: surgical clipping and angiological coiling. Respondents emphasized that the decision to continue treatment is made on an individual basis, especially in the case of continued complications.

#### 3.2.3. Outcomes

In addition to the use of standard outcome scale measures, clinicians reported that they depended on their experience to perform evaluations on an individual basis regarding the probable prognosis and complications. They assessed the outcome positively if the patient ultimately survived in a better condition than expected. An unfavorable outcome for clinicians was when the patient died or survived with severe disabilities after a long period of suffering. They also emphasized that “negative examples” remain longer in their memory.

*[Example of a former patient]* “*Where we didn’t see any chance that it would turn out well in any way […] after half a year we saw her for the first time, but she could already walk on her own. So yes, [she walked] with support. With a person support, but she has/she was cognitively/[…] probably not like before. But you could communicate with her and she had quality of life.*”

(CL02, 23)

#### 3.2.4. Everyday Life in the nICU

Clinicians described the daily work necessary to avoid complications and manage treatment. The interviews show some indications of increased psychological stress, for example, in the use of war imagery:

*[On having discussions with LOs:]* “*And you don’t have to fight with relatives alone, even if it [gets long and complicated], not alone, […] we don’t always have to be on the front lines, we can also hand it off [to colleagues].*”

(CL02, 81)

In terms of coping strategies, interviewees found discussions with colleagues (CL01, 104–111) beneficial. They also reported deriving satisfaction from seeing clinical improvement in their patients. CL02 (79) was encouraged in his work when he earned the trust of the affected person’s loved ones.

#### 3.2.5. Decision-Making

Clinicians spoke about the process and content of decision-making in the ICU; the benefits of advance directives; and the influences of interdisciplinary discussions and clarifications with the next of kin. Upon initial presentation, they stated that a decision is made regarding the initial treatment, either coiling or clipping. There are, then, many subsequent treatment decisions. Clinicians emphasized that in the nICU, there is a critical question of whether the patient would benefit more from sedation to avoid stress or whether the ability to perform close monitoring, which is impaired by sedation, is more beneficial.

“*I think the first big decision is you have to decide which direction [in treatment] you want to go. For example, can I extubate the patient? Or can I reduce sedation to the point where he’s awake? That would be the big goal. But is that good for the patient? So, is it more stress for the patient, which then again promotes complications, or is it good for the patient if we can assess him better?*”

(CL02, 37)

Ideally, a patient’s preferences should be assessed at the beginning of the hospital stay. The interviews suggested that this goal was not consistently met in reality. Often, the patient’s will was not considered until questions about palliative care were raised.

“*It is actually also part of the initial consultation [to ask] about the living will, and it should be said, depending on how the patient is doing, or how old he is, you already ask about the patient’s will. So, if they are severely affected and old, then of course you usually ask the next of kin what the patient would have wanted in the first place. If there is a living will, if they are younger and fitter, then this is also important and should of course also be made, but not necessarily in the first discussion.*”

(CL01, 43)

Clinicians reported that decision-making was not only influenced by medical evidence but also depended on the affected person’s values and the next of kin’s sense of self-efficacy in decision-making. Clinicians suggested that next of kin need to see patients to understand their condition and consider treatment options. Clinicians indicated that visitation bans during the first lockdown in the coronavirus pandemic strained the decision-making process.

“*At the lockdown last March, when the relatives were not allowed to come, I noticed that it was much more difficult to make them understand that you cannot discuss certain things because they did not understand certain things. The day before, the patient was fine. Then he fainted briefly and then they didn’t see him anymore. So, to speak. Suddenly you say, it’s not going to work out/we have to stop the therapy now in the worst case. And in between the two weeks that he lay here ventilated and so they did not realize it. I find when the relatives come to visit regularly, and just also see that the patient is not responsive, whatever else, or has stress, or is still intubated or/I find that/maybe also just the time duration to be able to accept that. That is very helpful.*”

(CL01, 161)

Even when next of kin were routinely present in the nICU, clinicians reported having to provide a lot of information and identified the importance of giving the next of kin as much time as possible to make decisions, since next of kin were often overwhelmed.

“*If the relatives are totally overwhelmed, then it often helps if you give them time. So, in the beginning it’s always a lot of information. Later, when they have visited [the hospital] several times, they also learn a little bit. So, then they see the change, or they see the possibility of giving the patient time.*”

(CL02, 67)

Clinicians also noted that the religious beliefs of the affected person and their next of kin would affect decision-making at the end of life. Clinicians reported that next of kin sometimes cited religious reasons for requesting life-sustaining treatment against medical advice. Clinicians said that they found it challenging when the next of kin insisted on artificial life support for religious reasons contrary to medical advice.

“*In this case [case description of a Hindu patient] it was said that he must not die on Fridays because that is somehow not good in Hinduism. […] Or simply in the Muslim culture it is very often that as long as medically everything can be done, it has to be done and that is also very difficult to talk about a change of therapy.*”

(CL01, 79)

One clinician suggested that religious families draw strength and hope from their faith (CL02, 68–75).

Clinicians reported that medical interventions and the evaluation of complications were conducted by an interdisciplinary team (CL01, 55), with the final treatment decision made by the directly responsible medical discipline. In consent discussions with next of kin, the clinicians reported that NoK were more concerned about the overall situation than about the specific treatment, such as whether to insert a feeding tube or move ahead with a tracheostomy:

“*These are minor operations [feeding tube, tracheotomy], […] usually the relatives are then already very/yes how should we say? Very tired. There are not many expectations. So, the expectation of an artificial feeding tube is now a different one. I think the relatives mostly have other concerns than now the feeding tube. I think they see it more like we do. It’s a purely technical issue. They don’t expect it to be a breakthrough recovery.*”

(CL02, 63)

For serious discussions and decisions such as end-of-life issues, clinicians reported that they must know the entire patient history and interdisciplinary strategies and, ideally, have already had contact with the next of kin (CL01, 53). Clinicians use a variety of strategies to reach out to next of kin:

“*Change the doctor who leads the conversation … with other words … time and conversations … try to repeat what was discussed*”.

(CL01, 73, 103)

### 3.3. Second Section—Main and Subcategories from the Perspectives of Those Directly Affected and Their Next of Kin

The interviews with those directly affected and their next of kin revealed insights into the disease experience, daily life with aSAH, decision-making values, outcomes, impacts on loved ones, and coping strategies. Seven main categories (see Table 3) emerged from the material and will be explained here and illustrated by verbatim quotes from the interviewees. The category of faith, religion, and spirituality was included after input from the clinicians’ interviews. The analysis revealed the identity category and subcategories, such as loss of autonomy, coronavirus, family disposition, and road traffic.

#### 3.3.1. Experience in Emergency Bleeding Situation

aSAH is characterized by severe headaches and loss of consciousness. Interviewees described the bleeding event as sudden and unexpected, most reporting that they knew something was wrong.

“*I was in the shower and suddenly my eyes went black, I had a severe, very severe headache, like an axe in the forehead. And gone. Suddenly gone. And, then I heard/in the next picture I heard voices. My wife, she reanimated me, so to speak. And I became wake again, and abnormally strong headache.*”

(AF08, 17)

Some of those directly affected (AF03, AF05, AF08) stated that they were immediately aware that their lives were in acute danger, while others (AF07, AF11) described becoming aware of the severity only later in recovery. They described near-death experiences (AF03, AF05, AF08) and stated that they became aware of their mortality due to the hemorrhage:

“*At one moment I just thought: Close your eyes, then it won’t hurt anymore. And I probably had the will to live.*”

(AF05, 28)

Accounts of the near-death experience were usually described in two main ways: sometimes the affected person described how he or she felt death (AF08), and sometimes he or she described knowing it was a life-threatening situation (AF03, AF05).

Those directly affected dealt with a variety of medical complications, such as increased cerebral pressures (AF03, 67, NoK04, 12), epidural hematoma (NoK04, 12), vasospasm (AF08,18), weight loss (AF08, 18), and cardiac problems (NoK09, 28). In the subcategory disorientation, respondents described states of altered consciousness or limited awareness of events.

“*Actually, it was not going so well for me. But I didn’t even realize that. And that is really an interesting process.*”

(AF03, 99)

The bathroom was a topic in almost all interviews. For the interviewees, this place became significant, often as the place of the initial bleed (AF03, AF07, Af08, AF19) but also as an important mark of ability when able to shower again (AF03, AF05).

#### 3.3.2. Diagnosis and Treatment

The stay in the ICU, at least two weeks in all cases, was challenging for all. The affected person often described coping with immobility, noise, and dependence.

“*And the worst thing for me in the ICU was the lying position. Having to go to the bathroom while lying in bed. Having these damn tubes everywhere, the monitors, the noises, […]*”

(AF03, 113)

The affected person often showed gratitude for the care received, which was evident in the experience with health professionals. Most often, the associations with health professionals were very positive:

“*And, so, the whole team was super. Super. Always being present, mega professional, mega competent. I’m speaking for the nursing staff now.*”

(AF08, 149)

Patients recalled personal interactions, such as a two-hour hair wash (AF11, 35). Some next of kin, though, expressed criticism, including that clinicians related to the affected person impersonally, identifying them as numbers, for example, or without enough sensitivity:

“*The doctors and nurses and so, always said, ‘ah yeah, have you been with seven or on eight?’*”

(NCC04, 85)

Rehabilitation was not only about physical recovery (NoK04, AF08, NoK09) but also about training cognitive abilities such as concentration (NoK04, AF05) and the stabilization of psychological problems (AF05). Reflections on experiences of this time varied greatly. Some reported that this time was valuable for them and was spent regaining capacity (AF03, 193). Others suffered from loneliness (AF11, 43), a lack of close support (AF08, 20), or the sense that their needs were not closely attended to, such as not being given sufficient pain medication (AF11, 43). Notable were statements of affected persons without physical limitations:

“*I didn’t have any big problems. It’s different when you can’t walk anymore or something. Whereas I would have liked that sometimes, almost. That sounds stupid now, that I would have had part of my body just/ and I would have gone to rehab and then learned to walk again. And I would probably have managed to do it again [to learn walking]. So, it would have been such a tangible process then. And with me, it was only neurological.*”

(AF03, 99)

These statements suggest that, in the minds of those affected, visible problems (motor deficits) were easier to manage than cognitive deficits.

Living arrangements following treatment depended on the state of health of the affected persons and the level of available support from loved ones. At the time of the survey, seven of nine individuals were living back in their households, one was in a nursing home (NoK04), and one was deceased (NoK06). In the support services category, respondents discussed how they were supported in the process. Depending on the affected person’s health status, support ranged from primary health care (NoK10, 45) and job coaching (AF08, 77) to work with physiotherapists and social workers (NoK04, 201).

In the subcategory loss of autonomy, respondents described limited self-determination and a lack of autonomy over their bodies. For example, respondents mentioned the complete loss of control of their bodies (AF03), being denied medical discussions with treating physicians (NoK04), paternalism (AF07), and medical coercion (NoK10). The words “prison”, “high-security wing”, and “isolation” were used to illustrate a state of restriction:

“*[When I think of the hospital,] prison always comes to mind. With the isolation cell.*”

(AF11, 45)

Some respondents had been hospitalized during the first wave of the coronavirus pandemic. The impact of the pandemic involved not only visiting restrictions (AF05, AF07, AF08, NoK09, AF11) but also difficulty understanding the condition (AF07, NoK09, AF11). Loved ones were limited in their ability to visit, and those directly affected registered the restriction as resulting in a lack of essential support. Loved ones also felt the disadvantage of limited visitation. In one example, a next of kin (NoK06) felt responsible for making palliative decisions on behalf of other family members who were not allowed to visit and who, therefore, felt unequipped to participate. The decision was, therefore, not made collectively. In addition, some respondents discussed the burden of getting COVID-19 itself (AF08, NoK09).

#### 3.3.3. Outcomes

With the wide range of outcomes (see Table 2), reflections varied when discussing how aSAH impacted respondents’ lives. Some described the experience of motor deficits (NoK04, AF05, AF08, AF11). In one severe case, the affected person was rendered immobile, bedridden, and highly sensitive (NoK04, 97). Those directly affected reported muscle loss due to prolonged bed rest (AF05, AF11). Many also experienced cognitive deficits that affected the ability to process information, including fatigue, poor concentration (AF08, 77), intense emotionality (AF11, 136), forgetfulness (AF07,86), impairments in logical thinking (NoK10, 41, 51), and anxiety (AF05, 41, AF03, 211). Some experienced psychological stress, which manifested as panic attacks (AF03, AF08) and anxiety (AF05) at the thought of rebleeding.

In terms of resuming functions, many mentioned the (dis-)ability to navigate daily traffic as a measure of their overall ability and progress. Some mentioned driving a car (AF05, 43, AF08, 199), and some mentioned riding a bike (AF07, 80, NoK10, 12). Not every affected person could return to their old occupation. Some of those affected described a process of reintegration; their ability to resume work is described in Table 2.

Those directly affected also reported that their quality of life increased in some important ways because they learned lessons from the crisis: they described giving more attention to their families (AF08, 28), taking more time away from work (AF11, 52), and spending more time on fulfilling life activities (AF07, 110). Depending on the type and degree of post-aSAH limitation, those directly affected revealed different coping strategies. Some adjusted their mindsets (“not taking everything so seriously” (AF03, 267)), while others restructured their daily lives:

“*In the beginning [after aSAH], I didn’t dare to be alone during the day. That became better with time. […] I always had the [apartment-] door open. I carry a phone [with me], so when I go to the toilet, the phone comes with me, when I go from the kitchen to the balcony, the phone comes with me […].*”

(AF05, 42)

#### 3.3.4. Impact on Loved Ones

aSAH impacted people in the affected person’s social environment in different arenas of life. Next of kin reported how the experience created a sense of shock (NCC06, NCC10) and a greater awareness of critical illness and mortality (NCC04, NCC09).

“*One is shocked [in the ICU] by the/yes simply by the view, by the helplessness of the pro/affected person, as well as the tubes and machines, what the intensive care unit naturally brings along. Yes, there is also the helplessness, of the patient or the helplessness of a self.*”

(NoK06, 52)

Fear of the future and additional loss, as well as regret, emerged as dominant emotions. One next of kin tearfully reported that she felt guilty being well because she knows how her mother suffers (NoK04, 175). Another regretted not accompanying his father to the palliative care unit (NoK06, 68).

Next of kin often described facing new roles following the event. In severe cases, they described assuming the role of legal surrogate (NoK04, 124). In another case, responsibilities and roles within the family were restructured:

“*She [my wife/NoK] has done so much for me. So many sacrifices. So much crying. For so long. A whole year, she was without me. She had the total lead in the family. So, the whole year: children, working, paying bills. So, it was also a financial crisis.*”

(AF08, 214)

Sometimes, next of kin reported conflicts with the affected person and other loved ones due to the burden of increased responsibilities, decision-making, and challenges with personality changes resulting from the brain injury. In the following example, the wife of an affected person described conflicts with her partner, who has cognitive deficits, because she had to offer considerable daily support, and yet he experienced frustration because he did not recognize his limitations. He was annoyed at being treated like a child, while she struggled to exercise patience.

*[Wife about her affected husband with severe cognitive deficits:]* “*He says, ‘You treat me like a five-year-old.’ I can’t go on. Really. I can’t discuss anymore. […] You see he’s not well, you try to help him, and then he comes up with something like this. He doesn’t notice anything, nothing.*”

(NoK10, 64)

Respondents mentioned children as an important factor. They were confronted with issues such as gaps in care (NoK04, AF08, NoK10), changes in parenting, challenging emotions (AF03, AF08, NoK09), and managing their children’s fears of loss (NoK04). Children were also perceived as valuable during difficult times:

“*Without children it would not have gone well at all. […] With a child you have to get up, you have to eat, you have to go out.*”

(NoK04, 189–191)

For those affected and unable to return to their previous jobs, loved ones faced additional financial and administrative burdens. A next of kin had to give authorities access to her finances to receive nursing-home funding (NoK04). One widow (NoK06) did not have access to joint bank accounts after her husband’s death because he had not granted her power of attorney during his lifetime. One wife (NoK10) was overwhelmed because she could not do all the tasks simultaneously: working full time, managing the household, raising the children, and structuring her severely disabled husband’s day.

Since aSAH can be genetically inherited, first-degree relatives reported having to deal with a possible familial predisposition. The interviews revealed ambivalence regarding how to interact with knowing that they may be at increased risk for aSAH. There were five reported ways in which respondents related to this family predisposition: repression (AF05), a sense of being overwhelmed and feeling concerned about lack of knowledge (NoK10), seeking medical advice (NoK09), having inner-family conflicts when one person wanted to have the other screened (AF05, NoK06), and preferring not to know (NoK06).

Next of kin talked not only about losses but also about lessons learned from the crisis. Above all, they integrated new knowledge into their everyday lives. Since, in one example, a next of kin (NoK04) has experienced how quickly life can end, she tries to avoid conflict in her daily life to be at peace with her loved one. The experience of a supportive community was also mentioned as an essential gain (NoK04, NoK06). One person described becoming more grateful for her own good health (NoK09).

“*I have become thankful again. […] What the body does for us without us doing anything. And that we rely on it.*”

(NoK09, 189)

The interviewees revealed what helps them cope with and adapt to new tasks and burdens. Strategies included spending time in nature or with children (NoK04, 225), faith community activities (NoK06, 138), yoga (NoK09, 141), and smoking pot (NoK09, 141).

#### 3.3.5. Identity

Many participants referred to their identity through statements describing themselves, a change in their physical composition, or indications of a personality change following the aSAH. Respondents differed in how they used comparisons to form their narrative identities. Some were deliberately removed from comparison making:

“*And I don’t question a lot of things in life. I accept things and I think that has helped me.*”

(AF03, 296)

Some used comparisons with others experiencing aSAH to describe their character and well-being (AF03, AF05, AF11). Some used comparisons with their lives before the aSAH to describe their present state (AF05, AF08, AF11).

Various indicators pointed to a change in the personality of the affected person, which was often experienced as a challenge for the next of kin:

“*That person [reference to the AF] is not coming back. She will no longer exist. […] she’s just someone different*”.

(NoK09, 153)

In some cases, changes occurred not only in the behavior of the affected persons but also in their physical composition. For example, their head shape changed after surgery (AF08, AF11), there was muscle loss and weight loss due to immobilization (NoK04, AF08), and medications caused changes in body structure (AF05).

#### 3.3.6. Faith, Religion, and Spirituality

Switzerland is a country with a fairly heterogeneous population and four national languages (German, French, Italian, and Romansh). In 2021, 26% of the permanent resident population were foreign nationals, and 39% had a migrant background (the top five: Italians, Germans, Portuguese, French, and Kosovars) [47]. The University Hospital Zurich is a public hospital. Most patients treated at the USZ reside in German-speaking cantons, particularly in the canton of Zurich or surrounding cantons. The permanent resident population (>15 years) of Zurich [48] indicated their religious affiliations (2020) as 26.4% Roman Catholic; 17.7% Protestant Reformed; 5.8% Muslim and Islamic communities; 0.9% Jewish religious communities; and 43.3% no religious affiliation.

Those affected and their next of kin discussed faith, religion, and spiritual thinking. The small sample showed a wide range of thoughts and affiliations. Respondents mostly came from or identified with Christian (AF05, NoK06, AF11) or Muslim (NoK04, AF08, NoK10) communities. One person considered himself an atheist (AF03). Respondents reported guardian angels (AF05), healing stones (NoK09, AF11), belief in rebirth (NoK09), conscious control of positive thoughts (NoK06, AF07), and in-depth discussion of philosophical questions (AF08). Two interviews showed the impact of religion, spirituality, and faith on decision-making: the respondents justified their preference not to learn about the potential risks of the disease or the rate of family predisposition for aSAH for spiritual reasons. For example, NoK06 distanced himself from medical assistance because of his belief that God would protect him:

“*The Bible says, ‘You can drink poison or stand on snakes and nothing will happen to us’, and that’s actually already taken the decision [to investigate family disposition] away from us. We say, ‘no, I don’t have to investigate that.’*”

(NoK06, 168)

In another case, an affected person (AF07) believed her good outcome was due to her good thoughts about her situation. In her mind, negative thinking, including knowledge of the severity of the disease, would have caused her disease to develop negatively. A Muslim family wished to continue therapy after an acute life-threatening deterioration. This decision was described as being based on personal values rather than religious grounds specifically. One respondent who identified as an actively practicing Christian explained his reasoning for choosing palliation based on information he received about the poor prognosis (NoK06, 176).

Religion was perceived as a resource when the faith community supported the grieving process (NoK06, 138), when it helped affected persons to focus on positive aspects of life (AF08, 240), and when it offered helpful approaches for managing difficult emotions (NoK09, 133). Belief in a higher power provided hope (AF05, 171).

#### 3.3.7. Decision-Making

Pathways to medical treatment: Those directly affected described how the decision-making process began before they entered the hospital. Some (AF07, NoK09, AF11) waited to see how symptoms would develop before they requested medical assistance, while, for others (AF03, NoK04, AF05, NoK06, AF08, NoK10), symptoms were so severe at the outset that emergency services were called immediately.

“*I was really screaming. I used to say, maybe they heard my screams miles away. I can’t describe the pain. Despite the pain, I noticed someone calling the emergency number.*”

(AF05, 28)

In all cases, next of kin were involved in calling for medical help or convincing the affected person to see a doctor.

Decision content: During the interview, those directly affected did not talk about being involved in shared medical decision-making. Either they did not know what treatments or interventions were performed (“I had no therapy” (AF05, 115)), or they were simply informed about what was to happen. (“He said, ‘you have to do this’” (AF07, 56)). One next of kin described a detailed process of shared decision-making around the choice of whether or not to go forward with a tracheostomy (NoK10, 115).

There were many uncertainties about prognosis in the acute phase of aSAH. For example, a next of kin (NoK10) indicated being unable to imagine how their futures would be affected. In particularly severe cases, the prognosis was sometimes clearer. In the following example, the next of kin describes being presented with information about probable disabilities, which was helpful for eventual decision-making.

“*[The AF was] in an epileptic state for so long that his brain was damaged to such a degree that they actually said he will, if he actually regains consciousness, he will be a high level of care. That means he will never get out of bed again.*”

(NoK06, 29)

With uncertain prognoses, next of kin described greater difficulty reaching decisions:

“*Yes, and suddenly they [the surgeons] called and said, ‘so and so’. […] yes, it was a heavy decision [re-operation in case of complication?]. So, we didn’t/one didn’t know, is it [the operation] successful or not? The doctor couldn’t really tell me what’s after either. And yes, they needed a simple decision.*”

(NoK04, 33)

None of those directly affected had a written advance directive (living will) at the time of their aSAH. Those directly affected could not recall previous conversations about their treatment preferences in the event of serious illness or injury. In the two severe cases (NoK04, NoK06), next of kin wished there had been a written advance directive because they felt responsible for making the decision and conflicted about how to reconcile their ideas about the affected person’s preferences with their own sense of what was right; both wished they had not had the burden of this decision-making.

“*And so, unfortunately, my parents didn’t have a living will. Mother [AF’s wife] was in such a state that she that they couldn’t actually make the big decisions. My siblings were not allowed to go to the hospital [because of the corona lockdown]. That is, in the end, it [the decision about the treatment goal] was actually up to me.*”

(NoK06, 29)

One person (AF07, 49) was grateful that she was not asked about her living will because she would have become aware of the seriousness of the condition. She reported feeling that, had she known, her anxiety would have negatively affected the healing process.

Respondents indicated various factors that influenced the process of decision-making. The interviewees often described periods of altered states of consciousness and disorienting emotions. Those directly affected reported memory lapses, distorted perceptions, and misperceptions about their health status.

“*He [the AF] also started/he got confused, everywhere. ‘Who are you? What’s your name?’. He didn’t know anything at all. ‘Do you have/how many children do you have?’ ‘Ten kids’ (showed ten fingers). ‘Oh dear.’ Then for me was really [hard].*”

(NoK10, 36)

One next of kin indicated there was difficulty with decision-making:

“*But due to the fact that my mother was actually not very capable of making decisions [about my father’s therapy] either, yes, the decision was more up to me.*”

(NoK06, 68)

Observing other patients was a reported factor in decision-making. In one example, an affected person decided against rehabilitation after seeing the experience of the woman with whom she shared a room:

“*So in the ward was a woman, where had already for two years rehab clinic […] and sometimes she sat at the table and painted mandalas. And I thought, ‘gosh, no, now I have to go to a rehab like that and paint mandalas.’*”.

(AF07, 33)

The willingness to be actively involved in the recovery process also depended on the person’s desire to leave the hospital (NoK10, 38), their feelings of isolation (AF08, 20, 25, AF11, 45), or their sense of not being understood (AF11, 45). Discussions with health professionals were perceived in a range of ways. In the best case, respondents described understanding what they were facing and making an informed decision:

“*It was always explained to me [on ICU] in detail what was happening. What to do. I also think it’s very important that/how should I say. Caring. But also very direct. So, nobody glossed over anything. Or so. This was very important for me. Or. No situation or anything was downplayed.*”

(AF03, 173)

In more negative reports, respondents stated that they felt that they were not being taken seriously (AF07, 42), could not evaluate the proposed therapies and possible consequences (AF11, 42), and made decisions that they regretted in retrospect (NoK04, 33). The following example illustrates that it was difficult for the affected person to understand the treatment because she could not understand the technical terms used:

“*They [clinicians] have talked with so technical terms (laugh) […] I have not known in the follow-up/after two months afterwards [after the hospital stay] that I also had a lung infection. Only because they always said ‘pneumology’ […] they always came with something to inhale, and I thought, ‘what are they doing for stress (laughter)?’*”

(AF11, 42)

Trust in clinicians appears to be an important basis for the ability of those affected and their next of kin to make decisions (AF07, 23) or to feel positive about the treatment process:

“*The doctor said, ‘we really didn’t give 100%, we gave 120%. We gave the best we could.’ […] I have seen as good as experience, positive. Said [to the doctor] ‘look, I’m leaving everything up to you. You know what is better, what, how. I trust you very much.’*”

(NoK10, 34, 114)

Next of kin also indicated there were additional discussions they wished had taken place. For example, one respondent expressed an explicit desire to have been better informed of the consequences of treatment interventions:

“*Or the shunt there (points to the neck), or they [the doctors] just said, ‘she needs that/ needs that’. So, okay, yeah, but just, no one ever really said if, […] we didn’t know, for example, that it stays throughout life, […] that we also have to keep going to control and justify and so on.*”

(NoK04, 135)

The retrospective evaluation of medical procedures tended to depend not on the specific intervention, but on the ultimate impact. For those who were relatively able to resume their previous lives, interventions were generally viewed more positively, whereas the worse the return to functioning, the worse the assessment of the medical procedure. In one case, the interviewee explicitly wished she had decided differently:

“*And if the doctors had somehow told me [NoK] more transparently there, ‘hey, look, she [the AF] will not come back after the surgery, the brain is too damaged’ or something, I might have made a different decision (crying), right? […] But I think if they would have told me more, ‘hey, look, it may be that it’s only 20% there now, where they say she’s coming back’, then I think I would have also, said, ‘okay, then they don’t operate’ (crying).*”

(NoK04, 115)

In the case of treatment discontinuation, one next of kin reflected that the decision had been reasonable, indicating that an outcome with many limitations would have been worse than death.

“*Five days between the event to the decision, right? And since one, yes, has felt at this point, that has been the right duration.*”

(NoK06, 176)

### 3.4. Third Section—Overview from the Comparison of the Results from Both Perspectives

The following table (Table 4) lists various issues related to decision-making and contrasts the clinician and AF and NoK statements. The key points will highlight the lessons learned.

Based on this comparison and the key points, we conclude that lay-oriented tools would be desirable to prepare people early on for possible upcoming interventions, decisions, and challenges. Such a tool could also support those who are uncertain about how to approach screening and the awareness of possible family predisposition.

## 4. Discussion

The present pilot study offers insight into the experience of aSAH, the decision-making process, and the consequences of aSAH from the perspectives of those directly affected, their loved ones, and treating clinicians in Switzerland. The comparison amongst the various perspectives with an emphasis on decision-making offers insights that might improve the care of those affected by aSAH.

The most important key points that can be drawn from Table 4 include the following: Decision-making in the face of prognostic uncertainty is the most challenging for all decision groups (CLs, AFs, and NoK). Prognostic descriptions must include probabilities and scenario descriptions. In disease progression, there is not just one preference-sensitive decision point, but rather multiple points in time depending on health progression, comorbidities, and complications. Decision-making with a longer consideration time is more manageable for all stakeholders than ad hoc decision-making. Awareness of these multiple potential decision points can lead clinicians to prepare AFs and NoK for upcoming decisions. Based on these findings, strategies should be implemented in NoK discussions, teaching sessions for health professionals, or decision aids.

The results indicate that clinicians feel limited in their present ability to make predictions about the disease progression, although, in some examples, clinicians seem to be quite certain about what will no longer be possible in the future. The clinician’s focus tends to be on treating the disease and making decisions based on the patient’s condition and guidelines. Those experiencing aSAH are often in an altered state of consciousness due to acute brain injury, the effects of medications, and delirium. Their loss of autonomy is particularly noticeable when they describe their time in the nICU as a prison. Those experiencing aSAH are often unable to make decisions for most of their nICU stay. Next of kin also describe the challenges of being unable to make decisions. This incapacity is evident when they hand over the decision to other family members or the professional ICU team. Aggravating the situation, those directly affected and their next of kin often did not understand the impact that the disease and related decisions would have on their future lives. This finding suggests that presenting outcomes as probabilities would be useful to inform decision-making when there is little certainty regarding future morbidity or functional status. We highlight the importance of refining conversations with next of kin to enable them to perform their tasks as best as possible. In the future, artificial intelligence (AI) might be able to support this process [18,49].

This triple challenge in decision-making—uncertainty, altered states of consciousness, and overwhelming demands—highlights the challenging situation of decision-makers. Not only do concrete treatment decisions have to be made [28,29], but the patient’s (presumed) will about his or her future quality of life must be centered. Rather than emphasizing individual interventions in advance directives, it may be more valuable to gather general guidance regarding under which conditions life is seen as worth living for the affected person.

Three opportunities to better promote fidelity to the patient’s will emerged. First, during the course of the disease, there are several preference-sensitive moments when the patient’s will should be clearly elicited and reevaluated. This could be better standardized. Second, NoK should be guided to work with likelihoods and probabilities. Third, advance directives should be geared towards delivering information that is helpful for clinicians and that patients can reasonably judge.

Few articles describe the affected person’s perceptions of the disease experience [20,21,24], and only some include the views of next of kin and medical personnel [32,33,34]. As described in the literature [20] and in the outcomes of this study, those experiencing aSAH suffer not only from cognitive and motor effects, such as fatigue, poor concentration, forgetfulness, and limited mobility, but also from changes in self-perception. The changed self-perception is not only caused by neurological changes and having been through such an experience but is also related to changes in physical composition due to altered body shape, muscle atrophy, or side effects of medication. Those directly affected also struggle with anxiety that manifests in panic attacks and limits in their daily lives. It is possible that those affected experience post-intensive care syndrome [50] due to the intensive care stay [51] and the near-death experience. These findings support the call for post-ICU care to better address psychological and emotional needs following such events, such as “better access to psychological support, including information, advice and peer or social support” [32]. These findings also support the need for research [20,26] into how next of kin manage such experiences.

The clinician interviews suggest that the burden may also be high for ICU clinicians, as manifested by high levels of psychological distress. The results suggest that prognostic uncertainty and challenging conversations with next of kin during therapy goals affect levels of distress. A review of burnout and ICU personnel [52] reports, according to the literature, that 6–47% of ICU staff experienced burnout. They identify risk factors such as young age, being single and childless, night shifts, and long working hours. Dealing with death and deciding to forgo life-sustaining therapies are also cited as risk factors for burnout. Another study on coping with poor or uncertain diagnoses in oncology [53] shows that neurologists and specialist nurses in neuro-oncology appear to have self-doubt because of a lack of data, highlighting the need for further studies and data collection. A quantitative study of high burnout risk and posttraumatic stress disorder in ICU personnel [54] found a preference for talking with colleagues and with people outside of work to reduce this risk. The authors recommend allowing more time for debriefing in difficult work situations. The results allow us to provide a deeper insight into the experiences of and challenges faced by those affected and their next of kin. By integrating the findings into the training of health professionals, as described in 2.6, we hope that they will conduct challenging conversations and decisions with more support and less distress.

In addition, some unexpected themes emerged during the pilot project that lend support for further elaboration: (a) Faith, religion, and spirituality: The potential religious or cultural context of the next of kin influences reactions to the loved one’s disease. When there are individuals with strong religious beliefs, especially from other cultures, the decision-making process is challenging for all involved. Based on the results, we hypothesize that the challenge between the different deciding parties increases when there is a wide divergence in values, which may be religious, spiritual, or cultural. Variations in end-of-life practices and decision-making in ICUs worldwide were observed [55]. This observation was also explored in a pediatric study in the Netherlands [56], which found that 26% of conflicts between parents and clinicians were based on religious reasons. American qualitative research [57] has shown that religious and spiritual influences on decision-making exist primarily in the areas of “hope and faith, God is in control, miracles, and prayer”. A neurological case study [58] described the refusal of surgery based on religious precepts in a neurological patient. We see the potential to systematically record faith, religion, and spirituality and cultural background in the patient record to explore differences in decision-making and disease progression. In expanding the pilot project, these categories should also be considered as selection criteria in the recruitment process. (b) Peer influence: Decision-making is influenced by other affected persons. The results suggest that sufferers compare their health status with that of peers. If they feel healthier than others or the peer treatment seems too easy (such as drawing mandalas in occupational therapy), they are less open to rehabilitation measures. A comparison with peer patients was identified in one study [20], but the literature search did not reveal further evidence to support this suggestion. However, one study [59] examining peers’ influence on moral preferences shows that peer observations influenced their behavior. (c) Family disposition: Relatives address family disposition and show different strategies to deal with this uncertainty. Although several studies positively evaluate systematic screening [60,61], relatives face many unanswered questions because they are not systematically informed about the disposition and possible screening. Based on these findings, we would advocate for the inclusion of the topic of “family disposition” when designing an interactive tool to support AFs and LOs.

### 4.1. Strengths and Limitations

The narrative DIPEx approach [39] gave interviewees flexibility in terms of the content, length, and structure of the interview. Participants spoke freely about feelings, needs, and daily challenges. Insights were gained into everyday realities and intimate thoughts. Respondents indicated that it was valuable to talk about their experiences with a researcher who is also a nurse in the ICU and appreciated feeling that they were contributing to the care of aSAH and improved decision-making processes despite the sometimes-distressing topic of discussion. After viewing the DIPEx website, one affected person said she was very positively and emotionally moved because she felt for the first time that others experienced the same challenges.

The structure of the study allowed for participant feedback to reach the ICU. For example, a next of kin responded that it would have helped to have materials to inform her children about the patient’s condition. In response, a children’s book about dealing with brain damage has been newly added to the waiting room of the nICU.

Our findings are linkable to current research, such as the national “Family Support intervention in Intensive Care Units” FICUS trial [62].

This work aligns meaningfully with the Swiss Academy of Medical Sciences’ 2019 Triple Aim framework, working toward the goal of integrating the public health perspective, individual perspective, and sustainability perspective into the health system [63]. This pilot study with qualitative data collection and analysis likely meets the criteria of credibility and transferability.

Based on the research design and choice of methods, we identified the following limitations:

- The pilot study did not reach theoretical saturation, with eleven participants from three groups, and the NoK’s relationship to the patient varied so much from one case to another that it was not possible to directly compare their experiences. For example, no comparisons were drawn when discussing the “impact on next of kin.”
- Moreover, it was broadly focused, so the results primarily indicate the different topics that should be further explored.
- Participants were sourced from only one institution with only German-speaking participants from three cantons. A larger sample with participants from across Switzerland and at least four spoken languages should be studied to represent the disease experience of aSAH in Switzerland more generally.
- The present target group excluded participants with other causes of brain damage, such as traumatic SAH or ischemic stroke; the findings might vary if the target group is expanded.
- During recruitment, those affected were not explicitly selected according to mental or cognitive deficits. Besides the interviews, there were no measurements available to record mental or cognitive deficits. Differences between participants are evident through self-report.
- Some potential interview participants declined to join the study because the topic was distressing. One person affected by aSAH who had significant cognitive deficits specifically asked the researcher to interview his wife instead of him. The views of such individuals were not included and may not have been reflected in the responses of study participants.
- aSAH has a long recovery time, and the time frame for this study presents challenges. For those interviewed too early, there may not have been sufficient time for recovery that would positively influence perceptions, whereas those interviewed later in the recovery process may not hold the memory of the initial experiences as clearly. Since respondents’ perspectives change over time—for example, one participant interviewed 19 months after the initial bleed stated that she experienced significant progress only in the last five months—the timing of the interviews may affect the findings.
- Due to the coronavirus pandemic lockdown, some interviews were conducted remotely; technical problems such as compromised sound quality occasionally led to the repetition of questions and answers, which may have affected responses.

### 4.2. Lessons Learned on Shared Decision-Making in aSAH after Perspective Comparison

Three opportunities to better promote fidelity to the patient’s will emerged.

(a) During the course of the disease, there are several preference-sensitive moments when the patient’s will should be clearly elicited and reevaluated. This could be better standardized.
(b) Next of kin should be guided to work with likelihoods and probabilities.
(c) Advance directives should be geared towards delivering information that is helpful for clinicians and that patients can reasonably judge.

## 5. Outlook

The planned expansion of the DIPEx project and the uploading of experience reports on DIPEx.ch (2023) will allow health professionals, AFs, and NoK to learn of other cases. We will provide the nICU with a flyer containing project information and the link to the DIPEx.ch website.

Future work might build on the present study by better highlighting when the patient’s (or NoK’s) input is most necessary. As the first step, we will publish a preference-sensitive decision-making timeline to better prepare health professionals as well as AFs and NoK for upcoming decisions.

## 6. Conclusions

aSAH was perceived as a life-threatening and life-changing event with various challenges. Physical, cognitive, and psychological deficits as well as the near-death experience caused affected persons to rethink their priorities. Depending on the severity, the impact also affected the lives of loved ones, as family roles must be renegotiated.

The results indicate that clinicians feel limited in their present ability to make predictions about the disease progression, although in some cases, they seem to be quite certain about what will no longer be possible in the future. In the first days following aSAH, prognostic certainty is very low, which poses challenges. Clinicians must explain what decisions need to be made under uncertainty and what the trade-offs are (treatment options, possible outcomes, likelihood of favorable outcomes, likely risks, and burdens, etc.). In cases of wide uncertainty about long-term prognosis and little information about the patient’s will, these findings suggest that treatments are usually continued (in dubio pro vita). The situation, though, needs to be regularly evaluated, and therapy goals should be regularly (re-)defined based on new available clinical information and, above all, based on the affected person’s assumed or documented will. Clinicians could be made more aware of the decisions that depend on patient preference and could be better supported in recognizing when to reevaluate patient preferences through (a) a conversation with the patient if he or she has the capacity, (b) an advance directive, or (c) the next of kin’s statements about the presumed will of the patient (in that order, at least according to Swiss law, and likely in many other countries). Managing treatment decisions requires a keen awareness of the preference-sensitive instances over the course of a patient’s treatment.

Probabilistic thinking is necessary in decision-making for which no certain statements regarding mortality or future morbidity/functional status can be made. This is demanding and requires guidance through excellent decision aids, drawing, e.g., on the possibilities of digitalization [64], and points to the need for evaluation if the care delivered is goal-concordant, i.e., corresponds to patients’ preferences [65]. The results show the need for a decision-making aid that might better prepare those affected and their next of kin for upcoming interventions, decisions, and challenges in a lay-oriented manner at an early stage.

## Figures and Tables

**Table 1:** Guides for semi-structured interviews for (1) clinicians and (2) affected persons and their next of kin.

Interview Guide Perspective 1—Clinicians	Interview Guide Perspective 2—Affected Persons and Their Next of Kin
1. Everyday insight Please describe how a patient with aSAH is cared for when they first come to your unit. What can they expect during the time in the hospital? Please explain as if I were a person from a different field. Describe a patient who has stuck in your memory. What are your tasks in the care of such patients? What is your first big decision when dealing with a new case of aSAH?	1. Beginning of the disease I would most like to hear about your personal experience. Please tell me about the day of the brain hemorrhage. What happened to you [your loved one] when you/they realized something was wrong [or when you heard what happened]? If you noticed something was wrong earlier, feel free to start the narrative at a different time.
2. Decision-making Can you tell me about the decisive moments for you that significantly influence the course of aSAH? What tools do you already use in your daily life that support your decision-making? How do you use them? What conditions do you need to enter into a conversation with surrogates to establish the goals of treatment and make decisions about possible palliation?	2. Hospital stay What memories do you have of your stay in the ICU? Please tell me about a situation that is stuck in your mind. Have you been asked to explain your will if you lose consciousness [your loved one’s will]? If yes, what helped you express this will [represent it in the best possible way]?
3. Interdisciplinary collaboration You don’t make the decisions alone. I am interested in how the decisions are made within the team. Who is involved in the decisions? Extending or discontinuing the therapy depends on which parameters?	3. Decision-making Please tell me how you experienced the conversations about upcoming treatments and therapies. What memories do you have of these conversations? At what moments did it become clear to you that this decision would impact the future?
4. Relatives For many interventions, you need the consent of the AF & NoK. Can you tell me for which interventions you obtain consent and how such discussions proceed? What questions and expectations do you face from the next of kin when you have discussions about treatment goals with them? How do you react when you notice that the next of kin are overwhelmed by the situation?	4. Development and changes What has changed in your life [your loved one’s life] because of the brain hemorrhage? (What was your life before the brain hemorrhage?) What gives you the strength to get through everyday life? What role does faith, religion or spirituality play in your life? What do you miss in your current life? What have you gained? What did you know about brain hemorrhage before this event? Maybe you have heard, read, or know someone with a similar diagnosis. What medical decision would you make now, with your current knowledge, if the brain hemorrhage were to happen today?
5. Final part of the interview I’m interested in how you deal with long decision-making processes and what helps you in your daily work. What points of contact or forums do you have where you can raise questions about challenging decisions? What else do you think is relevant to this whole topic? Do you have anything to add? Is there anything else that you think is still important that I haven’t touched?	5. Final part of the interview What else do you think is relevant to this topic? Do you have anything else to add? What advice would you give to someone arriving at the hospital with the same diagnosis? And what would you say to next of kin? Thank you very much. That was very interesting and informative. Is there anything else I haven’t touched on that you still think is important?

Query metrics and psychosocial data (age, occupation, diagnosis, hobbies, marital status) were additionally asked if not already mentioned during interview.

**Table 2:** Characteristics of interview participants.

Characteristics and Interview Parameters	CL ^1^ n = 2	AF ^2^ n = 8	NoK n = 4
Sex female			
Female	1.0	4.0	4.0
Age			
Median [Range]	40.5 [39–42]	51 [38–63]	38.0 [31–43]
Length of interview (minutes)			
Mean [Range]	36.4 [36–37]	86.2 [59–100]	80.5 [56–98]
Relationship to affected person (only NoK)			
Wife			1.0
Daughter			1.0
Son			1.0
Sister			1.0
Time between bleeding event and interview (months)			
Mean [Range]		17.0 [14–21]	
Treatment			
Coiling		2.0	
Clipping		3.0	
Unknown		3.0	
Glasgow Outcome Scale Extended (at Interview)			
1: Death		1.0	
2: Persistent vegetative state		1.0	
5: Moderate disability (lower)		1.0	
6: Moderate disability (upper)		1.0	
7: Good recovery (lower)		3.0	
8: Good recovery (upper)		1.0	
Residence (Canton)			
Zurich		6.0	
Lucerne		1.0	
Schwytz		1.0	
Migrant background ^3^			
Yes	2.0	4.0	2.0
Occupation before bleeding event		1.0	
Carpenter		1.0	
Quality manager		1.0	
Illustrator		1.0	
Geriatric nurse		1.0	
Mechanics		1.0	
Kitchen assistant		1.0	
Construction worker		1.0	
Farmer		1.0	1.0
Educator			1.0
Author			1.0
Production employee			1.0
Ability to work after bleeding event			
Self-employment		2.0	
Full-time		2.0	
Capacity building		1.0	
Unemployed		2.0	
Supported workshop		1.0	
Category not applicable (deceased)		1.0	
Place of living before bleeding event			
Together with family (partner/children)		5.0	
Alone		3.0	
Shared household with AF			1.0
Place of living after bleeding event			
Independent at home (with family)		2.0	
Independent at home (alone)		3.0	
Dependent at home (with family)		1.0	
Nursing home		1.0	
Category not applicable (deceased)		1.0	

^1^ No psychometric data were collected from clinicians to guarantee anonymization. ^2^ AF characteristics include both interviewees and references from interviews with their NoK; due to double case perspective, n (AF) ≠ n (interviews). ^3^ Not asked, interpretation based on biographical description, accent.

**Table 3:** Thematic focus on themes emerging from the interviews, divided into main categories (gray) and subcategories (white).

Clinicians		Those Directly Affected and Next of Kin	
Emergency care	Primary care Complications Prognosis	Experience in emergency bleeding situation	Bleeding event Near-death experiences Complications Disorientation Bathroom
Diagnosis and treatment	Diagnosis Treatment	Diagnosis and treatment	nICU Experience with health professionals Rehabilitation Living arrangements following treatment Supporting services Impact of the pandemic Loss of autonomy
Outcome	Assessing the outcome	Outcome	Motor deficits Cognitive deficits Psychological stress Navigate daily traffic Occupation Lessons learned from the crisis What helps?
Everyday life in the nICU	Daily work Increased psychological stress Coping strategies	Impact on loved ones	Shock Emotions New roles Conflicts When children are around Financial and administrative burdens Family disposition Lessons learned from the crisis What helps?
		Identity	Narrative identity Change in personality Physical composition
		Faith, religion, and spirituality	Faith, religion, and spiritual thinking Impact of faith, religion, and spirituality on decision-making Resources
Decision-making	Decision content Patient preferences Influence Interdisciplinary team Consent discussions	Decision-making	Pathways to medical treatment Decision content Prognosis Living will Influence Discussion with health professionals Evaluate medical procedures

**Table 4:** Synopsis of decision-making by contrasting two perspectives—clinicians and AFs and their NoK—including key points.

Topic	Clinicians	AFs and NoK	Key Points
First Decisions	Treatment coiling vs. clipping Stress reduction vs. neurological monitoring	Emergency call	The decision-making process already starts with the first symptoms.
Treatment Decisions	Ongoing decisions, such as weaning from the endotracheal tube or the insertion of an external ventricular drain, informed by guidelines and depending on the patient’s state	Therapeutic decisions are not recognized as such but perceived as information about the process. AFs are often unaware of what is happening in treatment.	Decisions seem to follow imperatively from a patient’s clinical development; choices may go unnoticed and not be discussed with NoK.
Prognosis	Compared with other forms of brain damage, the outcome of aSAH in the early phase of the disease is difficult to predict because of the high rate of complications and the complexity of interpreting imaging.	NoK want clarifications about the prognosis to inform decision-making. In addition, NoK cannot imagine how the event of aSAH will affect their future lives.	Prognostic uncertainty and lack of lay experience regarding future impacts are significant challenges in decision-making.
Forecast descriptions found to be helpful	Repetition of main statements; consulting with colleagues for other ways to explain the situation is useful.	Concrete scenario descriptions (“will never get out of bed”) and percentage of recovery chances (“20% … she’s coming back”) are perceived as useful.	Rather than pushing for a certainty that may not exist, working with likelihoods is useful.
Patient’s will	Clinicians emphasized the importance of the patient’s will, although their reports suggest that patient preferences are not always systematically elicited at the onset of treatment or during.	No study participant had a written advance directive. AFs could not recall any previous discussions about their presumed will in the event of incapacity. NoK found that they were responsible for determining treatment preferences.	There is potential for improvement in recording the patient’s will: (a) systematic elicitation of patient preferences and (b) emphasis to NoK that they are meant to represent the patient’s will, not their own.
Decisions that were regretted	Not applicable	Two examples of regret: (a) life-sustaining measures that resulted in severe disability and (b) behavior after the palliative decision (next of kin went home instead of spending last hours with his father)	Palliative decisions can be a source of retrospective regret.
Preference-sensitive moments	Initial consultation, after new findings (e.g., CT imaging), before invasive intervention (e.g., tracheostomy, PEG)	Bleeding event, initial consultation, after new findings, entry into post-inpatient care (e.g., rehabilitation, GP, nursing home)	At various stages, the patient’s preferences are relevant and should be reevaluated; knowledge and experience that are gained can inform advance directives.
Decision-making ability	Not applicable	AFs are often in an altered state of consciousness. NoK often suffer from shock and are overwhelmed.	The intensity of the experience impacts decision-making abilities of both AFs and NoK. This impairment of NoK is not considered in the Swiss law § 378 (ZGB).
Influence of coronavirus	Insight from the lockdown: discussions and shared decision-making with NoK are more effective and impactful if they happen in person.	Discussions and shared decision-making are more effective and impactful if they happen in person. NoK play an essential support role and impact the recovery process.	The experience with visitation restrictions highlights the importance of NoK’s presence. NoK benefit from direct engagement with the situation.
Other patients	Not applicable	Patients compare their health status with that of peer groups. This comparison influences their treatment preferences.	Health professionals should incorporate this knowledge into therapeutic discussions.
Time	It is important to communicate to AFs and NoK the time-sensitive nature of treatment decision-making.	NoK preferred having more time to make decisions and regretted some decisions made hastily.	The necessity of timeliness in decision-making should be emphasized. In case of foreseeable complications, NoK could be prepared in advance with descriptions of possible scenarios to aid advanced decision-making.
Values/worldview	Clinicians experience palliative decision-making with religious families as challenging. Medical decision-making is burdensome when the clinician and NoK have different values or worldviews.	There is some connection between a spiritual or religious worldview and decisions, and some decision-making seems more tied to cultural codes and family values.	There is a connection between worldview and decision-making; conflicts and challenges are noted on the clinician’s side when views do not align.
Communication	When gaining informed consent, clinicians report that NoK are more interested in the overall situation and outcome than in individual interventions or the details of procedures.	There is a wide range of perceptions about the quality of conversations with clinicians, including informative, reliable, clear, direct, vague, overly technical, and others.	The need for clear, forward-looking communication appropriate for the layperson should be emphasized. Advance directives should be geared toward delivering information that is helpful for clinicians and that patients can reasonably judge, such as overall goals of care or circumstances they want to avoid no matter what.
Family	Not applicable	First-degree relatives are not systematically informed about their genetic predisposition to aSAH. This topic elicits uncertainties, conflicts, and fears.	First-degree relatives might benefit from being systematically informed of their risk of familial disposition. People with a knowledge of their potential aneurysm can (a) be screened and treated and (b) write a focused advance directive.

## Data Availability

Some data presented in this pilot study will be published on the www.dipex.ch website in 2023.
